# Enhanced Barley Growth in Petroleum-Contaminated Soil Mediated by Xanthan-like Exopolysaccharide of *Xanthomonas translucens* TRK8

**DOI:** 10.3390/microorganisms14040937

**Published:** 2026-04-21

**Authors:** Ramza Berzhanova, Aisulu Zhuniszhan, Gulnur Tatykhanova, Sarkyt Kudaibergenov, Gulshara Abai, Alibek Kudabayev, Togzhan Mukasheva

**Affiliations:** 1 Department of Biotechnology, Faculty of Biology and Biotechnology, Al-Farabi Kazakh National University, Al-Farabi 71/19, Almaty 050040, Kazakhstan; ramza05@mail.ru (R.B.); aisulu_zhuniszhan@mail.ru (A.Z.); 2 Institute of Polymer Materials and Technology, Atyrau 1, 3/1, Almaty 050019, Kazakhstan; gulnur-ts81@yandex.ru (G.T.); skudai@mail.ru (S.K.); 3 BioClean LLP, Tole bi 289/14, Almaty 050031, Kazakhstan; abay.gk@mail.ru (G.A.); kudabaevalibek@mail.ru (A.K.)

**Keywords:** rhizosphere soil, oil contamination, EPS-producing strain, non-Newtonian rheology, emulsifying activity, seed priming, plant stress tolerance

## Abstract

Exopolysaccharides (EPS) represent an important tool for application in bio- and phytoremediation technologies due to their ability to enhance water and nutrient retention, support microclimate stability, and protect plants from environmental stress. In the present study, xanthan-like EPS produced by *Xanthomonas translucens* TRK8 was precipitated by ethanol and isopropanol, with the former yielding 9.2 g L^−1^ compared with 6.7 g L^−1^ obtained with the latter. The monosaccharide profile of the TRK8-derived EPS indicated a branched structure composed of rhamnose, mannose, glucose, and galactose residues, containing both α- and β-type pyranose units. The rheological properties of the studied EPS were compared with those of commercial xanthan at concentrations of 1–3 wt.%. Fitting the obtained data to the Ostwald–de Waele power-law model revealed that the flow behaviour index (*n*) values were below 1 (−0.338, −0.499, and −0.647, respectively), indicating shear-thinning behaviour (i.e., pseudoplasticity). The potential of the TRK8-derived EPS as a plant protection agent was validated by coating barley seeds with 2 wt.% EPS, resulting in a 28.6% increase in shoot length and a 64.7% increase in root length relative to the oil-stressed control.

## 1. Introduction

Extracellular polymeric substances (EPS) have long attracted interest due to their biodegradability, biocompatibility, and ability to thicken, gel, and stabilise emulsions. Microbial EPS are heterogeneous carbohydrate polymers produced by bacteria, yeasts, fungi, and microalgae. Under stressful environmental conditions, microorganisms synthesise EPS in the form of capsules or mucus layers, where they contribute to biofilm formation and environmental stress tolerance, thereby protecting cells from extreme conditions [1,2,3,4]. EPS-producing microorganisms are ubiquitous and can be isolated from diverse aquatic and terrestrial environments, including wastewater, marine water, soil, and various plant-associated niches, as well as from the gut microbiome, fermented foods, and extreme microbial habitats [5,6,7]. Well-known examples of EPS include xanthan produced by *Xanthomonas* species, dextran synthesised by various lactic acid bacteria, alginate from *Azotobacter* and *Pseudomonas*, curdlan from *Alcaligenes*, *Rhizobium*, and *Agrobacterium*, and gellan from *Sphingomonas* and *Pseudomonas* species [8,9,10,11,12].

Microbial EPS represent one of the most important groups of biopolymers and are widely applied across the pharmaceuticals, medical engineering, cosmetics, food, textile and oil industries, as well as in environmental protection. Most known bacterial EPS are highly water-soluble and exhibit stable rheological and emulsifying properties across broad temperature and pH ranges; in addition, they may function as antigens in Gram-negative bacteria [13,14,15]. Owing to their unique structures and tuneable properties, bacterial EPS possess considerable potential for numerous industrial applications, which has stimulated continuous interest in identifying new producers and novel EPS to develop functional materials with diverse uses.

The genus *Xanthomonas* comprises more than 20 species of phytopathogenic bacteria that infect a wide range of host plants and represent significant agricultural pests, frequently causing black rot, a common and destructive plant disease [16]. Most species within the genus are pathogenic to approximately 400 plant species [17]. The most extensively studied EPS from this group is xanthan gum, produced by *X. campestris* pv. *campestris*. Research on xanthan gum has been conducted in numerous laboratories, and its commercial production began in 1964. Many other *X. campestris* pathovars, including *phaseoli*, *malvacearum*, *carotae*, *citrumelo*, and *juglandis*, as well as additional *Xanthomonas* species such as *X. fragariae* and *X. oryzae* pv. *oryzae*, are also effective EPS producers [18]. Several other *Xanthomonas* species have since been identified as xanthan gum producers, including *X. gummisudans*, *X. juglandis*, *X. phaseoli*, *X. vasculorum*, *X. arboricola* pv. *juglandis*, *X. axonopodis* pv. *vesicatoria*, and various additional pathovars, such as *begonia* and *dieffenbachia* [19,20,21,22]. Multiple species, including *X. arboricola*, *X. axonopodis*, *X. vasculorum*, *X. citri*, *X. malvacearum*, *X. carotae*, *X. gummisudans*, *X. juglandis*, *X. fragariae*, and *X. phaseoli*, are suitable for industrial xanthan production; however, *X. campestris* remains the most widely utilised species [22,23,24].

In this study, a new EPS-producing strain, *Xanthomonas translucens* TRK8, was characterised using Fourier transform infrared spectroscopy, high-performance liquid chromatography, nuclear magnetic resonance spectroscopy, thermogravimetric analysis, and scanning electron microscopy. The emulsifying and rheological properties of the EPS were examined, and its potential applications in agriculture were assessed. The screening of microorganisms capable of producing polysaccharides with valuable functional properties, along with investigations of their physicochemical characteristics and potential uses, remains an active and relevant area of research. In light of the limited data available on xanthan-like EPS produced by *X. translucens* and its agronomic utility under petroleum stress, the xanthan-like EPS derived from the TRK8 strain may serve as an alternative biopolymer with distinct rheological properties and monosaccharide composition, thereby broadening the diversity of microbial EPS while contributing to stress mitigation, plant protection, and enhanced biodegradation efficiency.

As part of the Food and Agriculture Organization’s work on soil restoration [25], substantial areas of agricultural land have been reported to be contaminated with xenobiotics. Therefore, the identification and conservation of novel microbial strains with industrial potential, particularly those capable of synthesising biopolymers suitable for economically sustainable production, are of considerable importance. In this context, the exploration and application of local microbial strains producing environmentally beneficial compounds are especially relevant for Kazakhstan, where infrastructure for scaling up bioprocesses remains insufficiently developed. This limitation represents one of the key barriers to the transfer of technologies from laboratory research to industrial application. A significant proportion of exopolysaccharides used in industry are currently imported, increasing production costs and reinforcing technological dependence. The utilisation of local microbial strains for EPS production may therefore enhance synthesis efficiency while simultaneously reducing reliance on imported biopolymers and lowering cultivation costs, owing to the inherent adaptation of these strains to local environmental conditions.

The present study aimed to evaluate the following properties of xanthan-like EPS produced by mucus-forming bacteria isolated for the first time from the rhizosphere of yarrow growing in the Trans-Ili Alatau Mountains, *X. translucens* TRK8 [26]: (i) non-Newtonian rheology; (ii) gel formation; (iii) film formation; (iv) emulsifying activity; and (v) seed-coating performance. *Hordeum vulgare* L. seeds were used as a model organism to demonstrate the applicability of TRK8-derived xanthan-like EPS as an eco-friendly strategy to support phytotechnology in anthropogenically disturbed areas and agricultural systems in arid regions. In this context, the xanthan-like EPS was evaluated as a bio-coating material capable of supplying water to seeds and enhancing seed germination.

## 2. Materials and Methods

### 2.1. Study Object

*Xanthomonas translucens* TRK8 (*X. translucens* TRK8) was isolated from the rhizosphere of yarrow (*Achillea micrantha* Willd). Plant samples were collected in August 2023 during expeditions in the foothill and lowland regions of the Trans-Ili Alatau. A detailed description of the procedures used for the isolation and screening of EPS-producing strains has been reported previously by Zhuniszhan et al. [26]. For maintenance and EPS isolation, yeast extract–peptone–glucose (YPG) medium (g L^−1^) [27] was used, containing agar–agar (20.0), yeast extract (10.0), peptone (25.0), glucose (20.0) and molasses-based medium consisting of K_2_HPO_4_ (3.0 g) and molasses (45 g) [28], respectively.

Genetic identification of the selected strain was performed by 16S rRNA gene sequencing. Given that species of the genus *Xanthomonas* may not be reliably resolved on the basis of the 16S rRNA gene region alone, comprehensive cellular, morphological, and biochemical characterisation was additionally undertaken. The resulting cultural and phenotypic characteristics were compared with published taxonomic descriptions of *Xanthomonas* species. *X. translucens* TRK8 was characterised by Gram staining using the potassium hydroxide (KOH) method [29]. Oxidative–fermentative metabolism was determined according to the method of Hugh and Leifson [30]. For genomic DNA extraction, the strain was cultivated on LB agar plates for 48 h. Chromosomal DNA was isolated using the Wilson method [31]. The 16S rRNA gene was amplified from 1 μL of the DNA extract using oligonucleotide primers 8F (5′-AGAGTTTGATCCTGGCTCAG-3′) and 806R (5′-GGACTACCAGGGTATCTAAT-3′). Sanger sequencing of the amplified 16S rRNA gene fragments was performed at the National Centre for Biotechnology (Astana, Kazakhstan). The resulting 16S rRNA gene sequences were compared with reference sequences in the NCBI database using the BLASTn algorithm [32].

The strain was evaluated for pathogenicity by a certified laboratory of JSC “Nutritest” and was determined to be non-pathogenic to humans and animals (Certificate No. 3620, dated 19 October 2023).

### 2.2. EPS Isolation and Purification

Exopolysaccharides (EPS) were produced by inoculating 10 mL of bacterial suspension (cell concentration approximately 10^11^–10^12^ colony-forming units (CFU) mL^−1^) into 90 mL of molasses-based YPG liquid medium in 300 mL conical flasks (n = 3). Cultivation was performed on an orbital shaker at 28 ± 2 °C and 180 rpm for 72 h.

Following incubation, the culture broth was separated from bacterial cells using sterile 50 mL Falcon tubes (n = 6) by centrifugation at 4000 rpm for 30 min (centrifuge Eppendorf 5810, Wesseling-Berzdorf, Germany). The supernatant was collected, and chilled ethanol or isopropanol (3:1 ratio) was added to precipitate the polysaccharides. The mixture was maintained at 4 °C for 12 h to ensure complete precipitation. The precipitated material was washed twice with cold ethanol or isopropanol and recovered by centrifugation (at 5000 rpm for 20 min). Dialysis was performed via 12–14 kDa dialysis tubing with a molecular weight cut-off (MWCO) against deionised water (diH_2_O) for 7 days, with daily water changes. After dialysis, the purified solution was lyophilised using a FreeZone 2.5 L freeze dryer (−50 °C; Labconco, Kansas, MO, USA) to obtain dry polysaccharide powder [33].

### 2.3. Determination of EPS Chemical Characteristics

#### 2.3.1. Determination of Carbohydrate and Protein Content in EPS

For the determination of total carbohydrate and protein contents in the produced EPS, 4 mg of EPS was used per sample (n = 3). Total carbohydrate content was quantified using the phenol–sulphuric acid method, with glucose employed as the calibration standard [34]. Protein concentration in crude EPS extracts was determined by a colourimetric assay using a commercial BC-Analysis kit (Interchim, Montluçon Cedex, France), in accordance with the manufacturer’s instructions. Standard curves were prepared with bovine serum albumin (BSA) and cytochrome C. Measurements were carried out in a 96-well microplate, and absorbance was recorded at 562 nm [35].

#### 2.3.2. Fourier Transform Infrared Spectroscopy (FTIR)

The chemical structure of the EPS was analysed using FTIR spectroscopy. FTIR spectra of the exopolysaccharide were recorded using a PerkinElmer Spectrum One IR spectrometer (PerkinElmer, Springfield, IL, USA) equipped with an iD5 attenuated total reflection (ATR) accessory with a diamond crystal. Following acquisition of a background spectrum, 1 mg of EPS was placed directly onto the attenuated total reflectance (ATR) crystal and scanned over the range of 400–650 cm^−1^ at a spectral resolution of 4 cm^−1^, with an average of 28 scans per sample (n = 3). The resulting spectra were processed, plotted, and analysed using OriginLab 2021 v. 9.8 software.

#### 2.3.3. Proton Nuclear Magnetic Resonance Spectroscopy (^1^H NMR)

The main functional groups of EPS produced were identified by Resonance Spectroscopy (^1^H NMR). The EPS sample (10 mg) was dissolved in 0.7 mL of D_2_O in a 4 mL sterile test tube. ^1^H NMR spectra were recorded on a Bruker BioSpin Avance III NMR spectrometer operating at 500 MHz (Bruker, Billerica, MA, USA). Spectral analysis was performed using Bruker TopSpin™ 3.6.2 software at 80 °C, with 128 scans acquired per spectrum. All chemical shifts were expressed in parts per million (ppm).

#### 2.3.4. Gas–Liquid Chromatography (GLC)

The monosaccharide composition of EPS was determined by gas–liquid chromatography (GLC) following conversion to polyol acetate derivatives. Briefly, 0.4 mg of EPS (n = 3) was hydrolysed with 0.5 mL of 2 M trifluoroacetic acid (TFA) at 120 °C for 2 h. Hydrolysis was performed in screw-cap test tubes fitted with Teflon-lined caps using a laboratory heating block (Multi-Blok, Barnstead Lab-Line, Waltham, MA, USA).

Following hydrolysis, monosaccharides were reduced with sodium borohydride (NaBH_4_; 10 mg mL^−1^) in 1 M ammonium hydroxide (NH_4_OH) at 20 °C for 2 h. The reaction mixture was neutralised with 10% acetic acid in methanol and subsequently treated with methanol. After solvent removal using a rotary vacuum evaporator (RV 10, IKA, Staufen, Germany), acetylation was carried out with an acetic anhydride/pyridine (Ac_2_O/Py; *v*/*v* 1:1) mixture at 100 °C for 1 h.

Standard monosaccharides (rhamnose, fucose, xylose, arabinose, mannose, glucose, galactose, glucosamine, and galactosamine) were derivatised to their corresponding acetylated forms using the same procedure. Polyol acetates were analysed on a DB-5 capillary column (30 m × 0.32 mm, 0.25 μm; Agilent, Santa Clara, CA, USA) using a GC-2010 gas chromatograph (Shimadzu, Kyoto, Japan). Separation was performed under a temperature programme from 160 °C (1 min hold) to 250 °C at a rate of 7 °C min^−1^. The injection volume was 1 μL, the split ratio was 10:1, ultrapure nitrogen was used as the carrier gas, and the detector temperature was maintained at 270 °C.

In parallel, the monosaccharide composition of EPS (2 mg) was analysed following hydrolysis in 0.01 M sulphuric acid (H_2_SO_4_) at 80 °C for 2 h and subsequent neutralisation with 5 M sodium hydroxide (NaOH). Analysis was performed by high-performance anion-exchange chromatography with pulsed amperometric detection (HPAEC–PAD) using a Smartline 5000 system (Knauer, Berlin, Germany) equipped with a CarboPac PA20 column (4 × 250 mm; Thermo Fisher Scientific, Waltham, MA, USA).

#### 2.3.5. High-Performance Liquid Chromatography (HPLC)

The average molecular weight of EPS was determined by high-performance gel permeation chromatography (HPGPC) using a Smartline 5000 system (Knauer, Berlin, Germany) equipped with a PolySep-GFC-P 5000 column (7.8 × 300 mm; Phenomenex, Torrance, CA, USA) maintained at 30 °C and coupled to a differential refractive index detector.

Samples (n = 3) were analysed at a concentration of 1 mg mL^−1^. A 0.2 M sodium nitrate (NaNO_3_) solution was used as the mobile phase at a flow rate of 0.5 mL min^−1^. The system was calibrated with dextran standards of defined molecular weights (40, 70, 110, 229, 500, and 2000 kDa; Fluka, Seelze, Germany). The molecular weight of EPS was calculated based on the corresponding calibration curve [36,37].

### 2.4. Determination of EPS Physical Characteristics

#### 2.4.1. Thermogravimetric Analysis (TGA)

Thermogravimetric analysis (TGA) was conducted using a LabSys Evo TGA instrument (Setaram Technologies, Cesson-Sévigné Cedex, France). EPS samples (5 mg) in three replicates were heated from 25 to 800 °C at a rate of 10 °C min^−1^ under a nitrogen atmosphere. The resulting thermograms were processed, plotted, and analysed using OriginLab 2021 v. 9.8 software.

#### 2.4.2. Scanning Electron Microscopy (SEM)

SEM of the EPS was performed using a MIRA II LMU instrument (Tescan Group, a.s., Brno, Czech Republic) operating at 4 kV in secondary electron mode. Magnification ranged from 1000× to 20,000×. Samples (n = 3) were prepared by air-drying a drop of aqueous EPS solution (0.5 wt.%) on a silicon wafer at room temperature.

### 2.5. Determination of EPS Functional Characteristics

#### 2.5.1. Determination of EPS Rheological Properties

Aqueous solutions (1, 2, and 3 wt.%) of EPS and commercial xanthan gum (CXG; Meihua Holdings Group Co., Ltd., Langfang, China) were prepared (n = 3), and dynamic viscosity was measured using a ViscoQC 300 rotational viscometer (Anton Paar, Graz, Austria). The R5 rotor (spindle RH6) was used, and measurements were performed at rotation speeds corresponding to shear rates of 60–100 s^−1^. Rheological tests were conducted at 25 ± 2 °C in triplicate. The measurement range was defined using a shear rate control experiment, in which the maximum shear rate was set at 100 s^−1^.

The rheological behaviour was described using the modified Ostwald–de Waele power-law model (Equations (1)–(3)) [38,39]:

(1) $$\tau =K\gamma^{n}$$

(2) η=τ/γ

(3) $$\eta =K\gamma^{n-1}$$

where *τ*—shear stress; *γ*—shear rate; *K*—the consistency index; *n*—the flow behaviour index; and *η*—apparent viscosity.

Rheology of TRK8-derived xanthan-like EPS and CXG was described according to the following classification: *n* < 1 indicates shear-thinning behaviour; *n* = 1—Newtonian flow; and *n* > 1—shear-thickening behaviour [39].

#### 2.5.2. Determination of EPS Emulsifying Activity

The EPS emulsification index was determined according to a previously described method [40]. Briefly, 6 mL of selected hydrocarbons, crude oil (Balgimbaev field, Atyrau region, Kazakhstan) and high-viscosity semi-synthetic motor oil (Mobil; SAE viscosity grade 40) were mixed with 4 mL of EPS solutions at concentrations of 0.5, 1, 2, and 3 wt.%. The mixtures were homogenised by stirring for 5 min. Emulsifying activity (EA, %) was measured after 24, 48, and 168 h and recorded as EA_24_, EA_48_, and EA_168_, respectively. EA was calculated as follows:

(4) $$EA=\;\frac{Emulsion\;layer\;height}{Total\;mixture\;height}\;\times 100\%$$

#### 2.5.3. Determination of EPS Gel Formation Capacity

The gelling capacity of the EPS was evaluated by preparing cation-mediated gels using mono- and divalent cations (FeSO_4_ • 7H_2_O, CuSO_4_ • 5H_2_O, CaCl_2_ • 2H_2_O, and MgSO_4_ • 7H_2_O) and a trivalent cation (FeCl_3_ • 6H_2_O). For each test, 5 mL of EPS solution (0.5 wt.%) was mixed with 10 mg of the selected salt and stirred until the salt dissolved completely (n = 3). Gel formation occurred under neutral (pH 7) conditions. To assess the gel formation under alkaline (pH 8) conditions, 1 mL of 2 M NaOH was added to the EPS and salt mixture. Gel formation was evaluated visually, and gels were classified according to strength and homogeneity as follows: (+) for homogeneous gels that retained their structure during testing, (−) for homogeneous gels that did not retain their structure, and (−) also for heterogeneous gels [24].

#### 2.5.4. Determination of EPS Film Formation Capacity

EPS produced by the *X. translucens* TRK8 strain was dissolved in distilled water (dH_2_O) to obtain 1, 2, 3, and 4 wt.% solutions for film preparation. Glycerine (5 wt.%) and carboxymethylcellulose (CMC; 1.5 wt.%) were added to each EPS solution (n = 3). A 10 mL aliquot of each solution was poured into plastic Petri dishes and dried at 25 °C for 24 h. The average film thickness was 0.03 mm, measured using a digital micrometer (MC2-0025 mm, Standartex SRL, Sovico, Italy) as the mean of 5 measurements from 4 films (3 replicates).

Mechanical properties (tensile tests) were evaluated using a texture analyser TA.XTPlus (Stable Micro Systems, Ltd., Hamilton, MA, USA). Each film was cut into three rectangular strips (20 mm × 80 mm), mounted on tensile grips, and stretched at 0.5 mm s^−1^ in tensile mode until breakage. Young’s modulus (εm, MPa) was determined as the initial slope of the stress–strain curve within the elastic deformation range. Tensile strength at break (σ) was calculated as the ratio of maximum force to the initial cross-sectional area of the film. Elongation at break (mm) was determined as the ratio of the extension at break to the original length of the sample. Five replicates of each film type were analysed.

#### 2.5.5. Determination of Water Retention Capacity (WRC) and Moisture Content (MC)

TRK8-derived EPS and CXG (Meihua Holdings Group Co., Ltd., Langfang, China) were evaluated for water retention capacity (WRC) and moisture content (MC). To determine WRC, 0.02, 0.04, 0.06, and 0.08 mg of EPS were incubated in 2 mL of dH_2_O for 30 min at 27 °C (n = 3). Following water absorption, the wet mass of each sample (*W*_1_) was measured using an analytical balance (AS220.R2; RADWAG, Radom, Poland). The hydrated samples were subsequently dried at 80 °C for 10 h, and the dry mass (*W*_0_) was recorded [41]. WRC (Equation (5)) and MC (Equation (6)) were calculated according to the following formulas:

(5) $$WRC=\;\frac{W_{1}-\;W_{0}}{W_{0}}\;\times 100\%$$

(6) $$MC=\frac{W_{1}-W_{0}}{W_{1}}\times 100\%$$

where *W*_1_—the mass of the sample before drying; and *W*_0_—the mass after drying.

### 2.6. Seed Germination Assay

Barley seed cultivation was carried out in a gnotobiotic system. Barley seeds (*Hordeum vulgare* L., variety “Arna”) obtained from “Kazakh Research Institute of Agriculture and Plant Cultivation” LLP were surface-sterilised with 95% ethanol, followed by shaking in a 10% chlorhexidine gluconate (CHG) solution for 2–3 min [42], and subsequently rinsed with sterilised water. Sterilised seeds were subsequently soaked for 3–4 h in 2 wt.% EPS solutions or in dH_2_O, which served as the control. To simulate stress conditions, 2 wt.% crude oil (Balgimbaev field, Atyrau region, Kazakhstan; viscosity 40–60 mm^2^ s^−1^ [43]) was incorporated into the sand and thoroughly homogenised. Specifically, 3.5 mL of crude oil was added to 150 g of sterilised sand per pot and manually mixed with a glass rod for 5 min to ensure uniform distribution. Six barley seeds, pre-soaked in either a 2 wt.% EPS solution or dH_2_O, were sown per pot. Five pots were prepared per treatment, resulting in a total of 120 seeds (6 seeds × 5 pots per treatment × 4 treatments). Experimental pots were maintained in a plant growth chamber (Fujian Jiupo, Fuzhou, China) for 10 days at day and night temperatures of 25 °C and 18 °C, respectively, under a 12 h light/12 h dark photoperiod [44]. Murashige and Skoog (MS) medium (Sigma–Aldrich, St. Louis, MO, USA) was applied to each pot (10% *v*/*w*) following the established MS formulation [45] in accordance with the general sand–culture concept for roots anchored in sand wetted with mineral nutrients. After 10 days of growth, plants were harvested for further analysis. Root and shoot lengths were measured [44].

The following experimental treatments were established:

1. Control: dH_2_O-soaked seeds were sown in uncontaminated sterile sand to assess baseline growth in the absence of both oil and EPS.
2. Oil control: dH_2_O-soaked seeds were sown in sterile sand supplemented with 2 wt.% oil to assess plant growth under oil stress in the absence of EPS.
3. EPS control: 2 wt.% EPS-soaked seeds were sown in uncontaminated sterile sand to assess the effect of EPS under non-stress conditions.
4. Oil + EPS experiment: 2 wt.% EPS-soaked seeds were sown in sterile sand supplemented with 2 wt.% oil to evaluate the effect of EPS on plant growth under oil stress.

### 2.7. Statistical Analysis

Statistical analyses were performed using RStudio software (version 2023.06.0, build 421; RStudio PBC, 2023). One- or two-way analysis of variance (ANOVA) was used to identify statistically significant differences among experimental variants. When significant effects were detected by ANOVA, Tukey’s honestly significant difference (HSD) test was applied for pairwise comparisons. In case the Shapiro–Wilk test failed, a non-parametric Kruskal–Wallis test was applied to determine the significant difference, followed by pairwise comparison with ‘Bonferroni’ adjustment. Experimental variants were classified according to the test results using letter-based groupings in descending order at *p* < 0.05.

For seed germination assay, two factors (oil and EPS) were evaluated to influence barley growth.

## 3. Results

### 3.1. Microbiological and Molecular Profile of X. translucens TRK8

**Figure 1:**
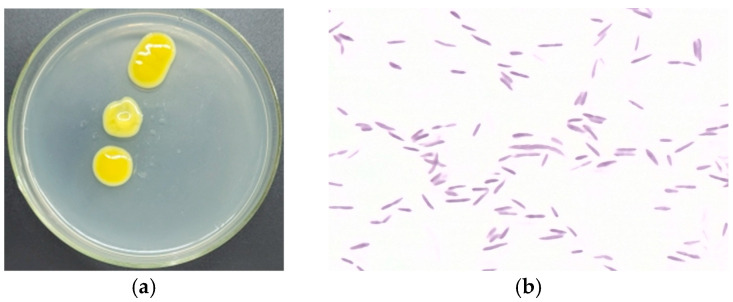
Isolation and characterisation of the studied strain. (**a**) Mucoid colonies of *X. translucens* TRK8; (**b**) micrographs of TRK8 cell morphology (magnification ×100).

Screening and selection of bacterial isolates for potential EPS production were based on the mucoid appearance of colonies grown on solid nutrient media [26]. The TRK8 strain exhibited the following cultural, morphological, physiological, and biochemical characteristics: on agar media, it formed non-fluorescent yellow, round, slimy colonies with smooth edges, a uniform oily consistency, and a convex elevation. Cells appeared as single, motile, Gram-negative rods without spore formation. TRK8 is an oxidase-negative, catalase-positive strict aerobe that forms capsules, liquefies gelatine, forms acid on sugars, but does not hydrolyse starch, and does not form poly-β-hydroxybutyrate. Representative images of TRK8 cells and colonies are shown in Figure 1.

Comparative analysis of the 16S rRNA gene sequence against the GenBank database, together with assessment of cellular morphology, colony characteristics, and biochemical properties, demonstrated that strain TRK8 was closely related to *X. translucens*. The 16S rRNA gene sequence of strain TRK8 was deposited in GenBank under accession number PP794702 (https://www.ncbi.nlm.nih.gov/nuccore/PP794702; accessed on 15 March 2026).

### 3.2. Chemical Profile of EPS Produced by X. translucens TRK8

**Figure 2:**
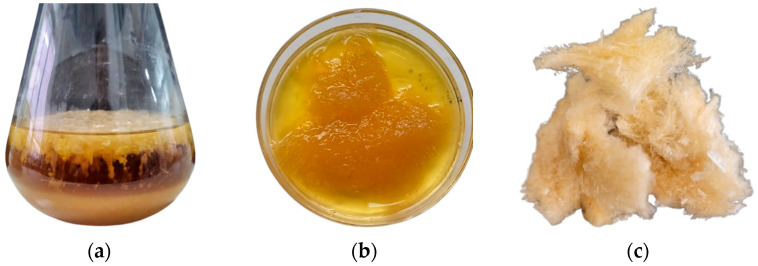
EPS production stages: (**a**) culture medium with EPS after fermentation; (**b**) EPS precipitated with ethanol; (**c**) lyophilised EPS.

The highest EPS yield (9.2 g L^−1^) was obtained when ethyl alcohol was used for precipitation and when molasses served as the growth medium, whereas the yield of EPS during isopropanol serving as a precipitation agent was 6.7 g L^−1^. The extracted and lyophilised EPS appeared as a creamy white powder (Figure 2). Chemical analysis showed that the EPS contained 78.1 ± 0.08% carbohydrates and 1.88 ± 0.01% protein.

**Figure 3:**
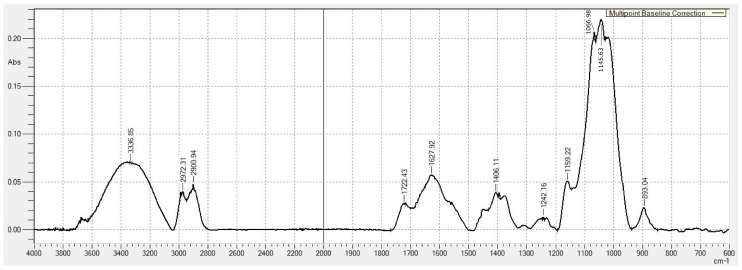
FTIR spectrum of raw EPS produced by *X. translucens* TRK8. Notes: The *x*-axis represents wavelength (cm^−1^), while the *y*-axis represents absorption (abs).

The chemical structure of the EPS was analysed using FT-IR spectroscopy (Figure 3). The EPS exhibited a broad absorption band at 3000–3600 cm^−1^, attributed to the stretching vibrations of the –OH groups. Additionally, the peaks at 2972 cm^–1^ and 2900 cm^–1^ were identified in all analysed films, corresponding to –CH and –CH_2_ groups stretching vibrations. The peak at 1722 cm^−1^ indicated the presence of C=O groups associated with glucuronic and pyruvic acids, whereas the absorption at 1627 cm^−1^ corresponded to COO^−^ groups. The band at 899 cm^−1^ suggested the presence of β-glycosidic linkages in the polysaccharide structure. The peak at 1406 cm^−1^ represented the deformational vibrations of methylene groups. An absorption band at 1242 cm^−1^ indicated asymmetric stretching of C–O–C bonds between carbohydrate residues and acetyl groups. The strong band at 1066 cm^−1^ corresponded to C–O–C stretching vibrations, which are typical of polysaccharides. Thus, the FT-IR profile indicates that the EPS produced by *X. translucens* TRK8 is likely structurally similar to xanthan gum.

Additional stretching absorption peaks for C–O were observed at 1020 cm^−1^, corresponding to C–O–H and C–O–C vibrations of the pyranose ring, indicating the presence of pyran ring conformations within the EPS structure [46].

**Figure 4:**
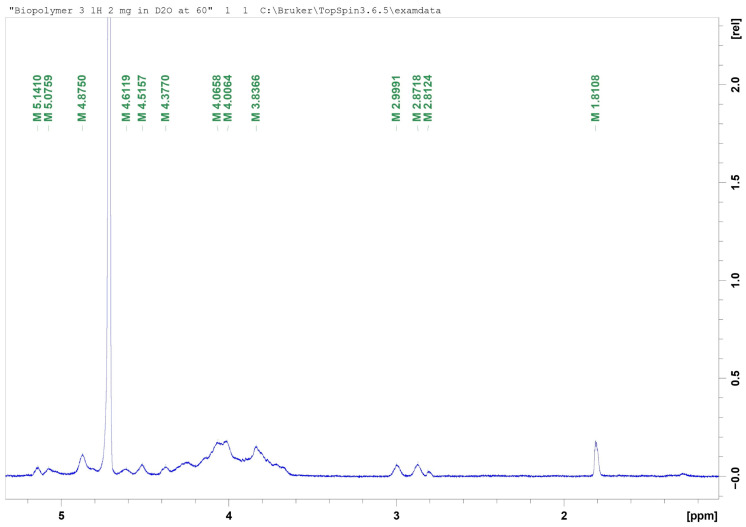
^1^H NMR spectrum of EPS produced by *X. translucens* TRK8. Notes: The *x*-axis represents chemical shift (ppm), while the *y*-axis represents a relative intensity in arbitrary units.

An intense signal at approximately 4.7 ppm was observed and corresponded to deuterium oxide (D_2_O). Resonances detected in the 3.8–4.2 ppm region were attributed to ring protons (H-2–H-6) of the sugar residues constituting the EPS structure. The signal at approximately 1.8 ppm was assigned to methyl protons of acetyl groups located within the side chains of the EPS. Weak signals in the 2.8–2.9 ppm region were attributed to protons adjacent to carbonyl or carboxylate groups, or alternatively to minor substituents within the polysaccharide backbone (Figure 4).

**Figure 5:**
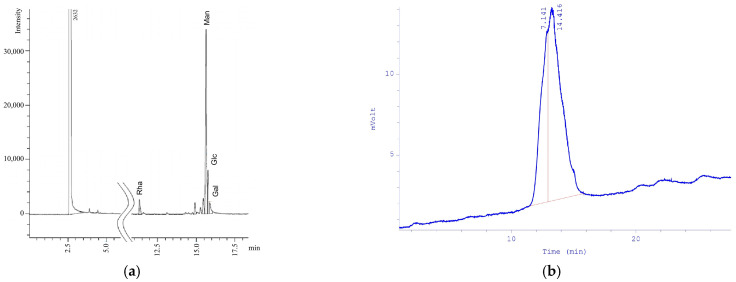
Monosaccharide composition of EPS determined by GLC analysis (**a**) and EPS homogeneity (**b**). Notes: The *x*-axis represents retention time (min), while the *y*-axis represents signal intensity (nC) for (**a**) and detector response (mV) for (**b**).

According to HPLC analysis, the monosaccharide composition of the EPS consisted of rhamnose (Rha), mannose (Man), glucose (Glc), and galactose (Gal) in the following molar ratio: Rha:Man:Glc:Gal = 1.21:12.6:3.59:1.00 (Figure 5a). HPLC showed that the molecular weight of the EPS was (1.0–1.2) × 10^6^ Da, being close to that of CXG (2.95 × 10^6^ Da). The single, symmetrical peak indicated that the purified *X. translucens* TRK8 EPS was homogeneous (Figure 5b).

### 3.3. Physical Profile of EPS Produced by X. translucens TRK8

**Figure 6:**
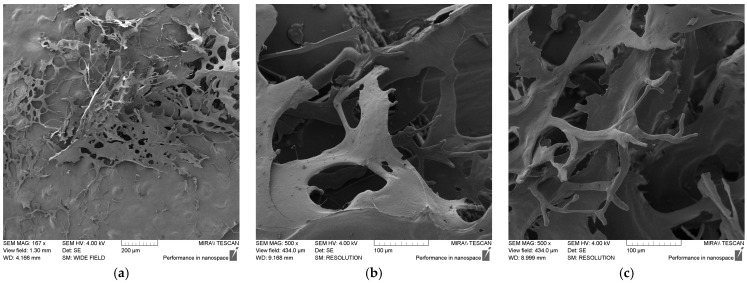
SEM images of lyophilised EPS at magnifications of 1.67 k× (**a**) and 5.00 k× (**b**,**c**).

The EPS produced by *X. translucens* TRK8 exhibited a mesh-like structure composed of dense, thick filaments with a rough and compact surface (Figure 6a). This morphology suggests that the EPS may possess favourable physicochemical properties, including high viscosity and strong water- and oil-retention capacities. At higher magnifications, additional microstructural features were visible (Figure 6b,c). The EPS displayed a rough surface and a highly branched network architecture, which likely contributes to its viscosity in solution.

**Figure 7:**
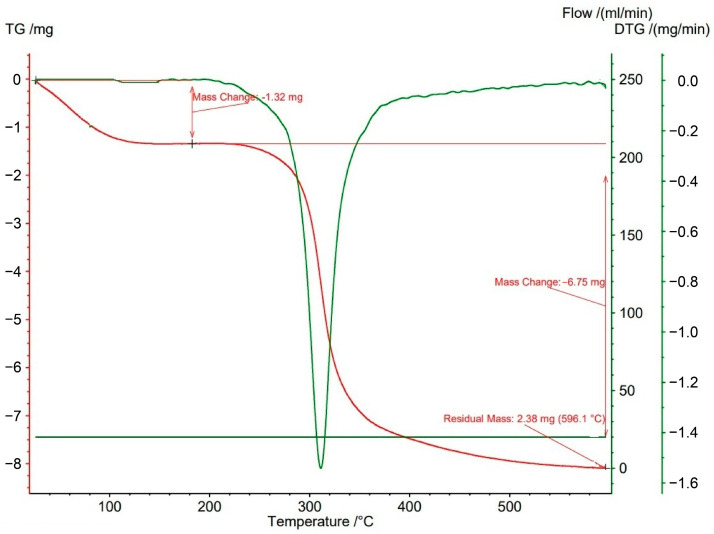
TGA curve of EPS produced by *X. translucens* TRK8. Notes: TG—thermogravimetric curve; DTG—derivative thermogravimetric curve.

A pronounced loss of EPS mass occurred at approximately 100 °C, corresponding to the evaporation of water molecules associated with the biopolymer (Figure 7). The recorded mass loss at this stage was approximately 13.2%. A subsequent sharp decrease in mass, amounting to about 67.5%, was observed at around 318 °C. This reduction is likely associated with depolymerisation and the breakdown of chemical bonds within the EPS structure. The substantial mass loss at this temperature indicates degradation of the main biopolymer component or significant chemical transformation. At the end of the thermal analysis, at 600 °C, 19.4% of the initial sample mass remained as ash.

### 3.4. Rheological Profile of EPS Produced by X. translucens TRK8

**Figure 8:**
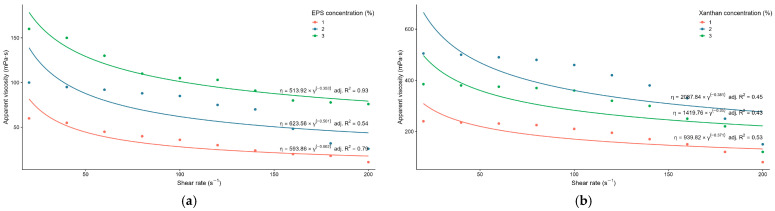
Dynamic viscosity of TRK8-derived xanthan-like EPS (**a**) and CXG (**b**) under increasing shear rate stress.

The rheological properties of the purified EPS were evaluated to assess its potential applications. The dynamic viscosity increased with increasing EPS concentration and depended on the applied shear rate. Aqueous solutions containing 2 and 3 wt.% EPS exhibited high dynamic viscosity and clear pseudoplastic behaviour, as viscosity decreased with increasing shear stress (Figure 8). The viscosity of EPS solutions recovered rapidly after the shear force was reduced, a property of considerable importance for practical applications.

The rheological properties of TRK8-derived EPS were evaluated in comparison with CXG. Dynamic viscosity increased with rising EPS concentration and was dependent on the applied shear rate. Aqueous solutions containing 1, 2, and 3 wt.% TRK8-derived EPS exhibited dynamic viscosity values ranging from 11 to 160 MPa • s (Figure 8a). Fitting the obtained data to the Ostwald–de Waele power-law model [38,39] demonstrated that, across the tested concentrations (1–3%), the TRK8-derived EPS exhibited pseudoplastic behaviour. Specifically, the flow behaviour index (*n*) values below 1 (−0.338, −0.499, and −0.647, respectively) indicated shear-thinning behaviour (i.e., pseudoplasticity) [39]. Notably, the viscosity data for the 1 and 3 wt.% EPS solutions showed a good fit to the Ostwald–de Waele model, with R^2^ values of 0.79 and 0.93, respectively. In contrast, only 53.5% of the viscosity dataset for the 2 wt.% EPS solution across the increasing shear rate range was explained by the Ostwald–de Waele model.

CXG solutions displayed higher dynamic viscosity (from 80 to 505 MPa • s) across all tested concentrations, with an approximately threefold reduction in viscosity observed as shear rate increased (Figure 8b).

The ability of the EPS to form gels was assessed in the presence of various multivalent cations (Mg^2+^, Cu^2+^, Ca^2+^, Fe^2+^, and Fe^3+^) under neutral (pH 7) and alkaline (pH 8) conditions (Table 1). Gel formation was evaluated based on strength and homogeneity: (+) indicates homogeneous gels that retained their structure, while (−) denotes homogeneous gels that did not retain their structure. The EPS formed strong, dense gels in the presence of Fe^2+^.

**Figure 9:**
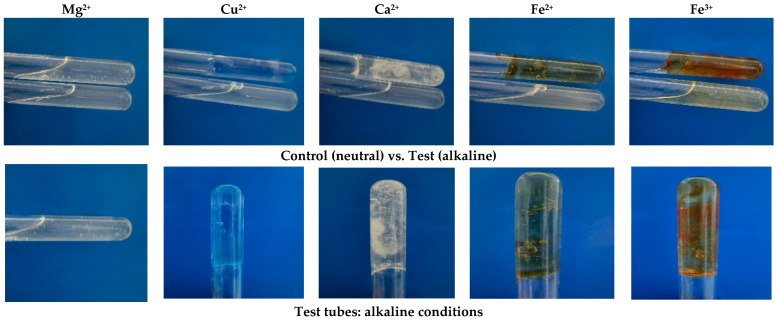
Gel formation of EPS produced by *X. translucens* TRK8 in the presence of various cations under standard and alkaline conditions.

For the *X. translucens* TRK8 EPS, weak gel formation was observed under standard conditions in the presence of tested cations. However, under alkaline conditions, EPS combined with Cu^2+^, Ca^2+^, Fe^2+^, and Fe^3+^ exhibited strong gel formation (Figure 9).

**Figure 10:**
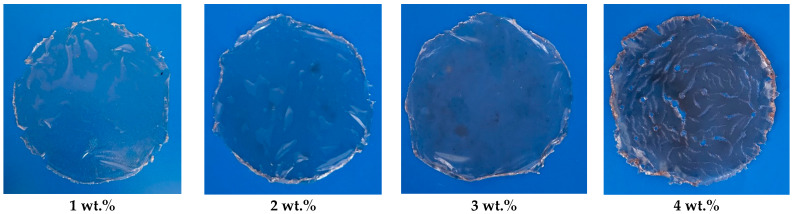
Photographs of films prepared using EPS produced by *X. translucens* TRK8.

The EPS films produced by the *X. translucens* TRK8 (Figure 10) demonstrated varying mechanical behaviour (Table 2). Films containing 3–4 wt.% EPS exhibited the highest tensile strength (20.0–25.7 MPa) and the greatest elongation at break (ε) (25.0–31.9%).

An increase in Young’s modulus from 70 to 90 MPa was observed, suggesting densification of the film structure, likely associated with the formation of a more compact polymer network and enhanced intermolecular interactions. The TRK8-derived EPS at a concentration of 2 wt.% exhibited the highest water retention, whereas lower polymer concentrations demonstrated greater overall moisture absorption capacity (Table 3). Increasing the EPS concentration to 3–4 wt.% led to aggregation and lump formation, which impaired uniform water retention.

Evaluation of the wet weights of EPS and CXG at different concentrations demonstrated a similar increasing trend with rising concentration (Table 3). In general, the wet weights of CXG were significantly higher than those of TRK8-derived EPS by 1.04–1.11-fold; however, at a concentration of 4 wt.%, the wet weights of EPS and CXG became comparable. This observation may be attributed to the significant differences in dry weights between EPS and CXG (1.30–1.84-fold).

The WRC of TRK8-derived EPS was determined to be 297%, 402%, 241%, and 200% at increasing concentrations, indicating a substantial capacity for water retention. In contrast, CXG exhibited lower WRC values of 183%, 168%, 152%, and 123% under comparable conditions. The WRC of TRK8-derived EPS was significantly higher than that of CXG, with 2 wt.% EPS demonstrating the highest value (402%). Meanwhile, the WRC of CXG peaked at 1 wt.% and remained at a comparable level at 2 wt.% (Table 3).

Moreover, the MC was consistently higher in TRK8-derived EPS than in CXG, by 1.17–1.24-fold (Table 3).

The emulsification activity of EPS (0.5–3 wt.%) was evaluated against different hydrocarbons. A suitable emulsifier should retain activity for at least 24 h after emulsion formation. EPS at concentrations of 2–3 wt.% exhibited the strongest emulsion-stabilising effect for E_24_ and E_48_, with values between 50 and 60% (Table 4). The emulsions remained stable for 168 h, showing slight improvement in E_168_ values.

### 3.5. Influence of EPS Produced by X. translucens TRK8 on Barley Seed Germination

**Figure 11:**
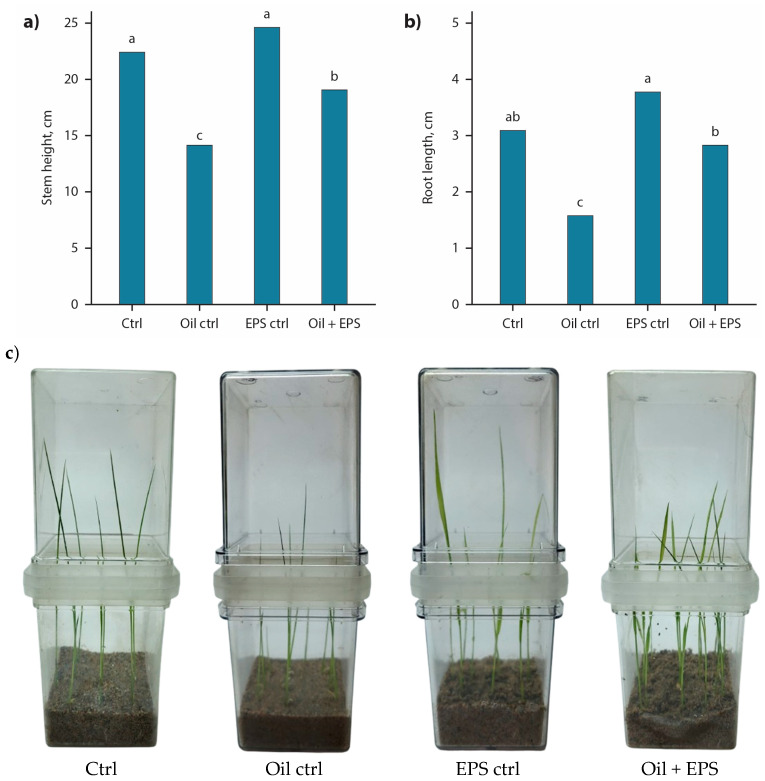
Effect of barley seed coating with EPS produced by *X. translucens* TRK8 (n = 5). (**a**) Stem length; (**b**) root length; (**c**) pots at the harvest. Note: Different letters within a single parameter (stem or root length) indicate statistically significant differences between treatments (*p* < 0.05).

High germination rates were observed across all experimental treatments, ranging from 97.8% to 98.6%. Evaluation of barley physiological parameters under oil-induced stress revealed a significant reduction in shoot and root length by 36.4% and 46.9%, respectively, compared with the control (Figure 11a,b). No statistically significant improvement in the measured parameters was observed in EPS-treated seeds under non-stress conditions relative to the control, although the recorded values were 9.09–15.6% higher. In contrast, barley seeds coated with 2 wt.% EPS derived from *X. translucens* TRK8 demonstrated markedly improved growth under oil-stress conditions. Total shoot length increased by 28.6% (n = 5, *p* < 0.05), and root length increased by 64.7% (n = 5, *p* < 0.05) compared with seeds grown in oil-contaminated substrate without EPS treatment. Under these conditions, shoot length reached values comparable to the control, whereas root length remained significantly lower (by 12.5%) than control levels. Furthermore, shoots of untreated plants exhibited chlorosis after 10 days of cultivation, as shown in Figure 11c. Collectively, these findings indicate that application of TRK8-derived xanthan-like EPS may represent a promising strategy for mitigating oil-induced growth inhibition in barley.

## 4. Discussion

### 4.1. Characterisation of X. translucens TRK8

Strains of the genus *Xanthomonas* inhabiting various plant species are widely recognised as potential EPS producers. *X. campestris*, *X. arboricola* pv. *juglandis*, and *X. axonopodis* pv. *vesicatoria* have been isolated from numerous host plants, including cabbage, kale, walnuts, peppers, mangoes, and tomatoes [3,5]. The isolation of xanthan-gum-producing strains is often carried out using cultivated plants of agricultural importance. In this study, the medicinal plant yarrow (*Achillea micrantha* Willd.) was used as a source for isolating EPS-producing microorganisms. The flora of the Trans-Ili Alatau is highly diverse, and among medicinal species, yarrow is widespread. For the first time, EPS-producing strains were isolated from the rhizosphere of *A. micrantha*, which is known to cause leaf damage in cereals growing in the foothill region. *Xanthomonas* colonies are typically yellow, smooth, and viscous [37]. Large, slimy, bright yellow colonies are generally associated with higher gum yields, whereas smaller colonies tend to be less productive [47]. Strain TRK8 exhibited intense colony pigmentation, consistent with elevated EPS production [47,48]. No observable changes in pigmentation were detected throughout the course of the study.

A total of 9.2 g L^−1^ of EPS was obtained from the *X. translucens* TRK8 using ethyl alcohol for polysaccharide precipitation. This yield is comparable to those reported for *X. campestris* pv. *mangiferaeindicae* 1230 (8.93 g L^−1^), *X. campestris* pv. *campestris* 254 (9.49 g L^−1^), and *X. campestris* pv. *campestris* 1078 (9.67 g L^−1^) [49], but lower than yields reported for other strains of *X. campestris* pv. *campestris* (11.0–15.3 g L^−1^) and *X. arboricola* pv. *pruni* 106 (16.0–19.5 g L^−1^) [50,51]. Considering study findings, although the EPS yield reported was at an intermediate level compared with those of other *Xanthomonas* species described in the literature, safety concerns related to the application of microbial products or microorganisms, particularly those associated with horizontal gene transfer, highlight the need to explore native microbial species. In this context, the present study represents one of the pioneering investigations in Kazakhstan, underscoring the novelty and relevance of the obtained results.

### 4.2. Structural and Chemical Characterisation of EPS Produced by X. translucens TRK8

Characterisation of the EPS produced by *X. translucens* TRK8 by FTIR, SEM, TGA, GLC, ^1^H NMR, and molecular weight determination revealed both similarities and differences compared with xanthan from other *Xanthomonas* species. FTIR spectra of EPS from *X. campestris* M28, *X. campestris* IBSBF 2103, *X. campestris* 1866, and *X. campestris* 1867 have previously been shown to display characteristic bands corresponding to hydroxyl (≈3200 cm^−1^), carbonyl (≈1400 cm^−1^), carboxyl (≈1600 cm^−1^), and acetal groups (≈1050 cm^−1^) [31,52,53,54]. The EPS produced by *X. translucens* TRK8 exhibited analogous bands and contained typical polysaccharide functional groups, including hydroxyl (OH), C–H, aldehyde (–CHO), and carbonyl (–C=O) moieties [55]. Consistently, ^1^H NMR spectra of the TRK8 EPS were in good agreement with those reported for xanthan gum [37,52].

Morphological analysis by SEM further supported a xanthan-like nature of the polymer while highlighting distinctive structural features. Xanthan from *X. campestris* PTCC 1473 has been described as fibrous and porous, which is typical for EPS of various *Xanthomonas* strains [27,53], whereas xanthan from *X. campestris* pv. *manihotis* 280-95 acquires a star-shaped, rough, compact surface after alkaline treatment [54]. In contrast, the EPS produced by *X. translucens* TRK8 displayed a naturally rough and compact surface without any chemical treatment, a morphology less commonly reported for *Xanthomonas* EPS [55]. This highly textured and branched microstructure suggests favourable physicochemical properties, such as enhanced viscosity and water- and oil-holding capacities [56].

Thermal analysis indicated that the EPS possesses good thermal stability. TGA curves showed an initial major mass loss around 100 °C, associated with the removal of bound water, followed by a second degradation step near 320 °C with over 60% mass loss. This behaviour is broadly comparable to xanthan, although the extent of weight loss was greater for the TRK8-derived EPS [56,57]. The relatively high degradation temperature supports its potential application as a film- or packaging-forming material [56].

The primary structure of xanthan from different *Xanthomonas* species typically consists of repeating pentasaccharide units composed of glucose, mannose, and glucuronic acid in molar ratios of approximately 2.8:2:2 or 2:2:2 [37,58]. In contrast, the EPS from *X. translucens* TRK8 contained mannose, glucose, rhamnose, and galactose. Xanthan is known to be a branched heteropolysaccharide in which a trisaccharide side chain (β-1,4-D-mannose, β-1,2-D-glucuronic acid, α-D-mannose) is linked to a β-1,4-glucose backbone via α-1,3 bonds [40,59,60], and its composition is strongly influenced by the producing strain and medium formulation [61].

Structural variability within xanthan-type polymers is well documented: rhamnose has been detected in xanthan from *X. campestris* pv. *juglandis* and *manihotis* [62,63]; *X. campestris* pv. *campestris* 8396 produced gum lacking glucuronic acid [64]; and pyruvate content may range from nearly zero to 8%, depending on the strain and environmental conditions [65]. The distinct monosaccharide composition of TRK8-derived EPS therefore fits within the broader structural diversity reported for xanthan-like polymers.

Xanthan typically displays very high molecular weights, in the range of 2.0 × 10^6^–10^7^ Da [66], which underpin its strong thickening ability, pronounced pseudoplasticity, and high stability to heat, acids, and alkalis, supporting its widespread use as a thickener, rheological modifier, stabiliser, and emulsifier [61]. In comparison, the EPS produced by *X. translucens* TRK8 had a molecular weight of 1.0–1.2 × 10^6^ Da, lower than most values reported for xanthan but still within the high-molecular-weight polysaccharide range [67].

Strain TRK8 was initially selected due to the high dynamic viscosity of its culture broth and stable emulsification index towards oils, indicating promising rheological behaviour of its EPS [68]. These properties were examined with a view to seed-coating and seed-protection applications.

Strain-specific characteristics and process conditions are known to modulate xanthan yield and quality; for instance, *X. arboricola* pv. *pruni* 31 produced high xanthan yield (26.4 g L^−1^) but with comparatively low viscosity [69]. Consistent with the behaviour of xanthan solutions, TRK8-derived EPS solutions exhibited non-Newtonian, shear-thinning (pseudoplastic) rheology, with viscosity increasing as EPS concentration increased [66]. The viscosity values for TRK8-derived EPS fell in the mid-range when compared with EPS from other *Xanthomonas* species: xanthan from *X. campestris* M28 displayed lower viscosity at 4 wt.% [70], whereas EPS from *X. campestris* pv. *campestris* 1866 and 1867 showed higher viscosity at 0.5 wt.% than that of TRK8-derived EPS [71,72].

The rheological behaviour of xanthan-type polysaccharides is largely governed by their chemical structure, molar mass, molecular conformation, and intermolecular interactions, all of which depend on the producing microorganism and growth substrate [70]. Uronic and acyl groups within the polymer facilitate the formation of physical cross-links through interactions with cations, promoting gel formation [73]. In line with this, TRK8-derived EPS formed strong gels under alkaline conditions, demonstrating its capacity to generate stable three-dimensional networks. Cation addition can also modify the polarity of EPS solutions and reduce polysaccharide charge, enhancing metal–polymer complex formation and increasing viscosity [74,75]. These features, together with the observed thermal stability and microstructural characteristics, indicate that EPS from *X. translucens* TRK8 has promising potential as a xanthan-like biomaterial for biotechnological and formulation applications.

### 4.3. Potential Applications of EPS Produced by X. translucens TRK8

Due to their structural diversity and functional versatility, polymeric materials are widely used for soil improvement. Microbial EPS are applied to soil in the form of hydrogels to enhance water retention capacity [76], improve soil structure [74], and support the remediation of soils contaminated with heavy metals or petroleum hydrocarbons [74]. Several studies have emphasised the potential of EPS as bio-emulsifying agents capable of enhancing the degradation and emulsification of hydrocarbons, with xanthan gum from *X. campestris* being one of the most effective examples [76].

Low-viscosity xanthan exhibits strong synergistic effects with other additives and high transparency, making it a promising candidate for agricultural and food industry applications [77]. According to the literature, xanthan is valued for its biodegradability, soil-reinforcement efficiency, film-forming capacity, good solubility in both cold and hot water, and its ability to form hydrogels [78,79].

Microbial EPS are known to protect plants from desiccation, thereby increasing plant vitality under adverse conditions [80]. In addition to mitigating drought stress, EPS facilitate bacterial attachment to plant roots. The higher water retention capacity of EPS also provides opportunities for improving soil properties, particularly through the mitigation of drought-induced stress. For example, xanthan-based hydrogel composites exhibit substantially enhanced moisture absorption and retention, together with reduced water evaporation rates. Bacterial EPS additionally contribute to the mechanical and structural stability of saline soils by promoting the formation of soil aggregates around plant roots [80]. Xanthan and alginate produced by *Xanthomonas* sp. and *Azotobacter vinelandii* have been shown to support aggregate formation and improve soil stability [79,80,81,82,83,84].

Areas contaminated with petroleum hydrocarbons represent critical environmental “hot spots” with significant ecological and public health risks. Bioremediation, an alternative to physical or chemical remediation approaches, relies on the bacterial capabilities to degrade hydrocarbons into simpler compounds (CO_2_, H_2_O, mineralisation in aerobic conditions) or to transform them into less toxic substances (incomplete degradation). Pre-sowing treatment of plant seeds with microbial biostimulants enhances germination, increases germination energy, and improves early seedling development under elevated salinity or pollutant load. Exopolysaccharide coatings have been shown to promote seedling and root growth in soils containing elevated levels of contaminants and salts (2–4 wt.%) [85].

Similarly, coating barley seeds with EPS from *X. translucens* TRK8 promoted seedling development under both oil-stress and non-stress conditions. The water retention capacity and protective properties of this EPS suggest the potential for a simple, inexpensive, and scalable seed-coating approach suitable for phytoremediation applications. Film coating is considered an effective and reliable approach for enhancing crop yields in the seed industry [84]. The EPS produced by the TRK8 strain forms mechanically robust films, which may increase the water-holding capacity of soil particles and contribute to the maintenance of soil moisture within the rhizosphere. Such a strategy could improve plant resilience and support the restoration of soils affected by petroleum pollution.

## 5. Conclusions

*Xanthomonas translucens* TRK8, isolated from the rhizosphere of yarrow, produced 9.2 g L^−1^ of EPS after 72 h of incubation. The EPS exhibited good thermal stability and formed stable gels under alkaline conditions. Its physicochemical properties were broadly similar to xanthan from *X. campestris*, although it differed in monosaccharide composition and molecular weight. HPLC and FTIR analyses indicated that the produced EPS was a heteropolymer with mannose being the predominant monosaccharide. Expanded polystyrene has demonstrated high water retention capacity, retaining approximately three times its own weight. Seed–EPS interaction studies showed that coating barley seeds with TRK8-derived EPS enhanced germination and improved seedling development in oil-contaminated soil.

The EPS produced by *X. translucens* TRK8 therefore demonstrates potential as a functional biopolymer for seed or root coating of phyto-agent plants in environmental remediation applications. Furthermore, the xanthan-like EPS derived from the TRK8 strain may serve as a plant protection agent to support agricultural development in arid and semi-arid regions. Future research should focus on optimising the EPS production capacity of the studied strain, improving the functional properties of the EPS, and extrapolating the sand–culture system experiments to real-case soil contamination scenarios involving hydrocarbons.

## Figures and Tables

**Table 1:** Gel formation under standard and alkaline conditions.

Conditions	Mg^2+^	Cu^2+^	Ca^2+^	Fe^2+^	Fe^3+^
Neutral	−	−	−	−	−
Alkaline	−	+	+	+	+

**Table 2:** Mechanical properties of EPS films produced by *X. translucens* TRK8 (n = 3). Note: Different letters within a single parameter (column) indicate statistically significant differences between EPS concentrations.

EPS Concentration, wt.%	Tensile Strength, MPa	Elongation at Break (ɛ), %	Young’s Modulus (E), MPa
1	11.3 ± 1.23 d	16.1 ± 1.05 d	70.2 ± 8.9 c
2	17.3 ± 1.32 c	19.2 ± 0.97 c	90.1 ± 8.2 a
3	20.0 ± 1.12 b	25.0 ± 1.12 b	80.0 ± 5.7 b
4	25.7 ± 0.87 a	31.9 ± 0.78 a	80.6 ± 3.4 b
*p*-value	<0.001	<0.001	<0.01

**Table 3:** Water retention capacity and moisture content of TRK8-derived EPS and commercial xanthan gum (n = 6). Different letters within a single parameter (column) indicate statistically significant differences between concentrations.

Concentration, wt.%	Wet Weight, mg			Dry Weight, mg		
	EPS	CXG	*p*-Value [Type]	EPS	CXG	*p*-Value [Type]
1	1.12 ± 0.05 d	1.24 ± 0.02 d	<0.001	0.26 ± 0.03 c	0.44 ± 0.04 c	<0.001
2	1.39 ± 0.01 c	1.52 ± 0.02 c	<0.001	0.31 ± 0.05 c	0.57 ± 0.05 b	<0.001
3	1.54 ± 0.04 b	1.60 ± 0.01 b	<0.01	0.46 ± 0.03 b	0.64 ± 0.04 b	<0.001
4	1.79 ± 0.07 a	1.76 ± 0.04 a	0.327	0.61 ± 0.04 a	0.79 ± 0.04 a	<0.001
*p*-value [Conc.]	<0.001	<0.001	−	<0.001	<0.001	−
**Concentration, wt.%**	**WRC, %**			**MC, %**		
	**EPS**	**CXG**	***p*** **-Value [Type]**	**EPS**	**CXG**	***p*** **-Value [Type]**
1	297 ± 19.0 b	183 ± 18.0 a	<0.001	76.3 ± 2.69 a	64.6 ± 2.48 a	<0.001
2	402 ± 25.9 a	168 ± 19.7 ab	<0.001	77.6 ± 3.82 a	62.5 ± 2.90 ab	<0.001
3	241 ± 17.0 c	152 ± 16.8 b	<0.001	70.2 ± 2.60 b	60.1 ± 2.41 b	<0.001
4	200 ± 7.43 d	123 ± 10.4 c	<0.001	66.1 ± 2.79 b	55.1 ± 2.22 c	<0.001
*p*-value [Conc.]	<0.001	<0.001	−	<0.001	<0.001	−

Notes: EPS—exopolysaccharide; CXG—commercial xanthan gum; WRC—water retention capacity; MC—moisture content; Conc.—concentration.

**Table 4:** Emulsifying activity (EA%) of TRK8-derived EPS against petroleum and engine oil at 24, 48, and 168 h.

Hydrocarbons	EPS Concentration, wt.%	EA_24_	EA_48_	EA_168_	*p*-Value [Time]
Oil	0.5	50.1 ± 0.12	53.4 ± 5.75	51.4 ± 5.69	0.700
	1	55.2 ± 5.89	55.7 ± 4.94	57.5 ± 4.86	0.857
	2	56.0 ± 0.06	54.7 ± 6.41	57.4 ± 2.28	0.721
	3	57.4 ± 5.69	60.1 ± 5.18	63.3 ± 4.16	0.431
*p*-value [EPS concentration]		0.217	0.525	0.065	−
Engine oil	0.5	51.4 ± 5.75	56.0 ± 7.16	58.8 ± 5.92	0.399
	1	60.3 ± 7.64	53.4 ± 5.75	61.1 ± 6.09	0.346
	2	57.7 ± 5.46	61.8 ± 6.53	61.4 ± 5.98	0.669
	3	57.4 ± 2.28	63.0 ± 6.08	63.7 ± 6.75	0.355
*p*-value [EPS concentration]		0.321	0.194	0.818	−

## Data Availability

The original contributions presented in this study are included in the article. Further inquiries can be directed to the corresponding author.

## Abbreviations

The following abbreviations are used in this manuscript:

ATR	Attenuated total reflection
BC	Bicinchoninic acid
BSA	Bovine serum albumin
CMC	Carboxymethylcellulose
CXG	Commercial xanthan gum
EA	Emulsifying activity
EPS	Extracellular polymeric substances
FTIR	Fourier transform infrared (spectroscopy)
GLC	Gas–liquid chromatography
HPLC	High-performance liquid chromatography
^1^H NMR	Proton nuclear magnetic resonance
SEM	Scanning electron microscopy
TGA	Thermogravimetric analysis
WAC	Water absorption capacity
WL	Water loss
